# Seropositivity for *Helicobacter pylori* and hepatobiliary cancers in the PLCO study

**DOI:** 10.1038/s41416-020-0961-0

**Published:** 2020-06-29

**Authors:** Rishi Makkar, Julia Butt, Wen-Yi Huang, Katherine A. McGlynn, Jill Koshiol, Michael Pawlita, Tim Waterboer, Neal D. Freedman, Gwen Murphy

**Affiliations:** 1 Division of Cancer Epidemiology and Genetics, National Cancer Institute, National Institutes of Health, Bethesda, MD USA; 2 Infections and Cancer Epidemiology; Research Program Infection, Inflammation and Cancer, German Cancer Research Center (DKFZ), Heidelberg, Germany

**Keywords:** Risk factors, Cancer epidemiology

## Abstract

*Helicobacter* has been suggested to play a possible role in hepatitis, gallstones, and hepatobiliary tumours. We assessed whether seropositivity to 15 *H. pylori* proteins was associated with subsequent incidence of 74 biliary tract and 105 liver cancer cases vs. 357 matched controls in the Prostate, Lung, Colorectal, and Ovarian Cancer Screening Trial (PLCO). Odds ratios and 95% confidence intervals were computed by conditional logistic regression after adjustment for known hepatobiliary cancer risk factors. *H. pylori* seropositivity was not associated with either biliary tract (1.76, 0.90–3.46) or liver cancer (0.87, 0.46–1.65). CagA seropositivity was associated with both endpoints, although the latter association was not statistically significant (biliary tract: 2.16, 1.03–4.50; liver cancer: 1.96, 0.98–3.93) and neither association was statistically significant after correcting for multiple comparisons. Together, these results suggest possible associations between *H. pylori* and hepatobiliary cancer and suggest the value of future studies investigating the association.

**Trial registration number**: NCT00339495.

## Background

*Helicobacter pylori* is a known risk factor for peptic ulcer disease and gastric cancer. Previous studies have suggested a role for *H. pylori* and other *Helicobacter* species in the incidence of hepatobiliary cancers.^1–3^ Cross-sectional studies have found evidence for *H. pylori* in liver^4–6^ and biliary tissue^7^ as well as in bile.^8,9^ Animal studies have shown that *Helicobacter* infections can cause hepatitis, gallstones, and liver cancer.^10–13^ Yet, few prospective studies have been conducted. We previously observed an association between *H. pylori* and subsequent incidence of biliary cancer (odds ratio (OR): 5.47, 95% confidence interval (CI): 1.17, 25.65) in the Finnish Alpha Tocopherol, Beta Carotene Cancer (ATBC) Prevention Study,^14^ with associations also observed for seropositivity to the UreaA, Omp, HP0231, and HP0305 antigens.

Liver and biliary cancers are a leading cause of cancer-related death and *H. pylori* is treatable. Thus a causal association between *H. pylori* and hepatobiliary cancers would have important public health implications.

Here we evaluate whether our previous findings in ATBC replicate in the US population.

## Methods

### Prostate, Lung, Colorectal, and Ovarian Cancer Screening Trial (PLCO)

Study participants were selected from the PLCO trial^15–17^ conducted to assess whether screening exams reduce mortality for prostate, lung, colorectal, and ovarian cancers. One hundred and fifty-five thousand participants aged 55–74 years were randomised between 1993 and 2001 to either the screening or the control arm. Non-fasting serum samples were collected from screening arm participants and stored at −70 °C. Cancer diagnoses were ascertained through annual questionnaires and confirmed by medical record review.

### Nested case–control study selection

Cancer cases through 2013 with available baseline serum were selected. The study included biliary tract cancers (gallbladder: International Classification of Diseases (ICD)-O-2 C23.9, extrahepatic bile duct: ICD-O-2 C24.0, ampulla of vater: ICD-O-2 C24.1, unspecified site: ICD-O-2 C24.8, C24.9) and liver cancer (hepatocellular carcinoma: ICD-O-2 C22.0, intrahepatic bile duct: ICD-O-2 C22.1). Control participants had available serum and were alive and free of hepatobiliary cancer at the time of case diagnosis. Cases were matched, pairwise, to two controls on age (+/−1 year) and sex. Controls were measured in the same batches as matched cases, except one control where the laboratory measurements failed.

### Laboratory analysis

Seropositivity to 15 specific *H. pylori* antigens was evaluated as previously described^18,19^ using a validated assay.^19,20^ *H. pylori* positivity was defined as being seropositive to ≥ 4 antigens, as in previously published studies.^20^ Performance across blinded replicate quality control (QC) samples was excellent. Six of the antigens displayed 100% concordance (GroEL, UreA, NapA, catalase, HcpC, Omp), three antigens displayed 99% concordance (CagA, VacA, Cad), and six antigens displayed between 90 and 97% concordance (Cag delta, HpaA, HP0231, HyuA, CagM, HP0305).

Assays for hepatitis B virus surface antigen, hepatitis B core antigen, and antibody to hepatitis C virus were performed as described previously^14^ with perfect concordance in QC samples with known viral status.

### Statistical analysis

Conditional logistic regression models (STATA v. 14.0, College Station, TX) were used to calculate ORs and 95% CIs, with adjustment for known and suspected risk factors (see Table 1, footnote).

**Table 1:** Odds ratio (OR) and 95% confidence intervals (CIs) for seropositivity to *H. pylori*^a^/CagA and risk of hepatobiliary cancers in the Prostate, Lung, Colorectal, and Ovarian Cancer Screening Trial (PLCO).

Serostatus	Total subjects	*H. pylori* seropositivity^a^				CagA seropositivity			
		Case +/−	Control +/−	OR (95% CI) (unadjusted)	OR (95% CI) (fully adjusted)^b^	Case +/−	Control +/−	OR (95% CI) (unadjusted)	OR (95% CI) (fully adjusted)^b^
Hepatobiliary cancer	536	92/87	162/195	1.31 (0.89, 1.92)	1.14 (0.73, 1.79)	71/108	94/263	1.96 (1.31, 2.94)	1.96 (1.21, 3.18)
Biliary tract cancers	221	35/39	53/94	1.64 (0.90, 2.99)	1.76 (0.90, 3.46)	25/49	32/115	1.96 (1.02, 3.76)	2.16 (1.03, 4.50)
Gallbladder	72	10/14	16/32	1.48 (0.51, 4.31)	1.56 (0.43, 5.66)	9/15	13/35	1.57 (0.57, 4.33)	1.75 (0.49, 6.30)
Extrahepatic bile ducts	75	14/11	19/31	2.17 (0.77, 6.09)	2.94 (0.87, 9.89)	7/18	11/39	1.40 (0.46, 4.29)	1.55 (0.42, 5.68)
Ampulla of vater	59	9 /11	16/23	1.13 (0.35, 3.70)	1.71 (0.30, 9.82)	7/13	8/31	3.39 (0.64, 17.93)	10.86 (0.87, 135.49)
Liver cancer	315	57/48	109/101	1.11 (0.68, 1.83)	0.87 (0.46, 1.65)	46/59	62/148	1.96 (1.17, 3.28)	1.96 (0.98, 3.93)
Intrahepatic bile ducts	39	5/8	9/17	1.18 (0.30, 4.63)	1.15 (0.27, 4.96)	5/8	4/22	2.91 (0.68, 12.45)	3.14 (0.59, 16.73)
Hepatocellular carcinoma	276	52/40	100/84	1.10 (0.65, 1.88)	0.93 (0.45, 1.93)	41/51	58/126	1.84 (1.07, 3.20)	1.99 (0.88, 4.50)

^a^*H. pylori* seropositivity is defined as positivity to ≥4 *H. pylori* antigens.

^b^Full adjustment included: cigarette smoking status (never, current, former), total cigarettes per day, alcohol consumption (<1 drink, 1–3 drinks, >3 drinks), college education, BMI (kg/m^2^), diabetes, gallbladder stones or inflammation, liver comorbidity (hepatitis or cirrhosis), hepatitis B, hepatitis C. Collinear variables and variables predicting outcomes perfectly were not included. Participants with missing data for variables adjusted for in multivariate models were included in the analyses with a flagged variable indicating missing data.

The study was performed in accordance with the Declaration of Helsinki.

## Results

No significant differences in baseline characteristics, by *H. pylori* serostatus, were noted except that controls positive for *H. pylori* were older, more likely to have diabetes, and more likely to have not provided information about their alcohol use (Supplementary Table 1).

Baseline *H. pylori* seropositivity was 47% in participants developing biliary tract cancer vs. 36% in matched controls and 54% in liver cancer cases vs. 52% in matched controls (Table 1). By cancer subsite, seropositivity for cases compared to controls was as follows: gallbladder 42% vs. 33%, extrahepatic bile duct 56% vs. 38%, ampulla of vater 45% vs. 41%, intrahepatic bile duct 38% vs. 35%, and hepatocellular carcinoma 57% vs. 54%. Seropositivity to *H. pylori* was not associated with either biliary tract (OR: 1.76, 95% CI: 0.90, 3.46) or liver cancer (OR: 0.87, 95% CI: 0.46. 1.65) overall or by subsite: gallbladder (OR: 1.56, 95% CI: 0.43, 5.66), extrahepatic bile duct (OR: 2.94, 95% CI: 0.87, 9.89), ampulla of vater (OR: 1.71, 95% CI: 0.30, 9.82), intrahepatic bile duct (OR: 1.15, 95% CI: 0.27, 4.96), or hepatocellular carcinoma (OR: 0.93, 95% CI: 0.45, 1.93).

Seropositivity for the CagA antigen, however, was associated with hepatobiliary cancer (OR: 1.96; 95% CI: 1.21, 3.18; Table 1), with increased risk of biliary tract cancer (OR: 2.16; 95% CI: 1.03, 4.50) and a similar, though not statistically significant increase in the risk of liver cancer (OR: 1.96; 95% CI: 0.98, 3.93). Two additional *H. pylori* antigens were associated with subsequent incidence of biliary tract cancer (Supplementary Table 2): GroEL (OR: 2.10, 95% CI: 1.00, 4.40) and HP0305 (OR: 2.21, 95% CI: 1.04, 4.69), but not liver cancer. None of these associations were significant after Bonferroni correction for multiple comparisons.

## Discussion

In this nested case control study, baseline seropositivity to *H. pylori* was not associated with either biliary tract or liver cancer, although the OR for biliary tract cancer was above one (1.76, 95% CI: 0.90, 3.46). Baseline seropositivity for CagA, on the other hand, was associated with biliary tract cancer with a similar odds ratio for liver cancer that was not statistically significant. Seropositivity to two additional antigens, GroEL and HP0305, was associated with incidence of biliary tract cancer but not of liver cancer.

In our prior study,^14^ we observed an association between baseline *H. pylori* seropositivity and subsequent incidence of biliary tract cancer (OR: 5.47, 95% CI: 1.17, 25.65). In this study, the OR was >1 (1.76, 95% CI: 0.90, 3.46) but not statistically significant. The random-effects summary OR from these two studies was 2.49 (95% CI: 0.89–6.91).

However, associations for individual antigens largely did not replicate between the ATBC and PLCO studies. In ATBC, but not in the current study, seropositivity to UreaA, Omp, and HP0231 were associated with biliary tract cancer. In contrast, CagA and GroEl were associated with biliary tract cancer in the current study but not in ATBC. Seropositivity to HP0305 was found to be associated in both the studies. In light of such inconsistencies and concerns about multiple comparisons, results for individual antigens in the current study should be interpreted cautiously. In addition to chance, differences between studies may reflect differences in populations, lifestyle, bacterial strains, and other factors.

All together, these results provide rationale for additional studies, particularly as both studies were relatively small and conducted in substantially different populations. Whereas ATBC had a high background prevalence of *H. pylori* infection (91.5%), prevalence in PLCO was much lower (47.4%). In addition, ATBC was undertaken among Finnish male smokers aged 50–69 years in the 1980s, whereas PLCO is a US study of men and women that began about a decade later and includes non-smokers.

The strengths of our study include a prospective design, detailed adjustment for potentially important confounding variables, and inclusion of both men and women. Limitations include a relatively small number of cancers and a population that was restricted mainly to white persons.

## Conclusion

In summary, results from the current study are supportive of possible associations between *H. pylori* and hepatobiliary cancer, indicating the value of future studies.

## Supplementary information

Supplementary tables 1-2

## Data Availability

Data from the Prostate, Lung, Colorectal and Ovarian (PLCO) Cancer Screening Trial is distributed using: https://biometry.nci.nih.gov/cdas/plco/.
