# Supplementary material for: Seropositivity for Helicobacter pylori and hepatobiliary cancers in the PLCO study
Source: Br J Cancer. 2020 Jun 29;123(6):909–11. doi: 10.1038/s41416-020-0961-0 (PMC7493958; doi:10.1038/s41416-020-0961-0)
Supplement: Supplementary file 1 — Supplementary tables 1-2 [file 41416_2020_961_MOESM1_ESM.docx]

**Online Supplementary Information:**

**Supplementary Table 1: Descriptive Characteristics of Controls from the PLCO Cohort by *H. pylori* Serostatus^1^**

| **Characteristic** | ***H. pylori* – (N = 195)** | ***H. pylori* + (N = 162)** | **P Value** |
| --- | --- | --- | --- |
| Age, yrs, mean, SD | 63.57 (5.39) | 65.09 (5.35) | 0.008^2^ |
| Sex |  |  |  |
| Male | 139 (71.28) | 119 (73.46) | 0.21^3^ |
| Female | 56 (28.72) | 43 (26.54) |  |
| Smoking Status, n, (%) |  | | |
| Never | 90 (46.15) | 72 (44.44) | 0.84^3^ |
| Current | 17 (8.72) | 17 (10.49) |  |
| Former | 88 (45.13) | 73 (45.06) |  |
| Smoking Pack, days, mean, (SD) | 1.37 (1.59) | 1.42 (1.59) | 0.77^2^ |
| Alcohol Consumption, n, (%) |  | | |
| 0 Drinks per day | 40 (20.51) | 35 (21.60) | 0.76^3^ |
| < 1 Drinks per day | 102 (52.31) | 91 (56.17) |  |
| 1-3 Drinks per day | 36 (18.46) | 24 (14.81) |  |
| > 3 Drinks per day | 17 (8.72) | 12 (7.41) |  |
| Missing Alcohol Data, n, (%) | 7 (2.48) | 20 (7.87) | 0.004^3^ |
| College Education, n, (%) | 114 (58.46) | 92 (56.79) | 0.75^3^ |
| Body Mass Index, mean, kg/m^2^, (SD) | 27.53 (4.77) | 27.61 (5.10) | 0.88^2^ |
| Diabetes, n (%) | 11 (5.64) | 19 (11.80) | 0.04^3^ |
| Gallstones, n, (%) | 17 (8.76) | 14 (8.70) | 0.98^3^ |
| Liver Cirrhosis or Hepatitis, n, (%) | 3 (1.54) | 5 (3.11) | 0.32^3^ |
| **Serum Markers** | | | |
| Hepatitis B Positive, n, (%) | 10 (5.13) | 12 (7.41) | 0.37^3^ |
| Hepatitis C Positive, n, (%) | 1 (0.51) | 4 (2.47) | 0.12^3^ |

^1^ *H. pylori* seropositivity is defined here as positivity to ≥4 *H. pylori* antigens

^2^ P values were calculated by Students *t* test

^3^ P values were calculated by χ^2^ test

**Supplementary Table 2: Odds Ratios (OR) and 95% Confidence Intervals (CI) for Seropositivity to *H. pylori* Antigens and Risk of Biliary and Liver Cancer in the Prostate, Lung, Colorectal, and Ovarian Cancer Screening Trial (PLCO)**

| **Antigen** | **Biliary Cancer** | **Liver Cancer** |
| --- | --- | --- |
| *H. pylori*^1^ |  |  |
| Case +/- | 35/39 | 57/48 |
| Control +/- | 53/94 | 109/101 |
| *Unadjusted* | 1.64 (0.90, 2.99) | 1.11 (0.68, 1.83) |
| *Fully Adjusted^3^* | 1.76 (0.90, 3.46) | 0.87 (0.46, 1.65) |
| GroEL- Chaperonin GroEL |  |  |
| Case +/- | 32/42 | 56/49 |
| Control +/- | 49/98 | 106/104 |
| *Unadjusted* | 1.65 (0.86, 3.17) | 1.13 (0.70, 1.81) |
| *Fully Adjusted^3^* | **2.10 (1.00, 4.40)** | 0.86 (0.45, 1.65) |
| UreA- Urease alpha subunit |  |  |
| Case +/- | 25/49 | 44/61 |
| Control +/- | 49/98 | 88/122 |
| *Unadjusted* | 1.02 (0.55, 1.87) | 1 (0.63, 1.60) |
| *Fully Adjusted* | 1.19 (0.60, 2.37) | 0.73 (0.38, 1.39) |
| HP0231- Hypothetical protein |  |  |
| Case +/- | 22/52 | 37/68 |
| Control +/- | 37/110 | 81/129 |
| *Unadjusted* | 1.25 (0.65, 2.38) | 0.85 (0.51, 1.42) |
| *Fully Adjusted* | 1.43 (0.67, 3.06) | 0.98 (0.50, 1.93) |
| NapA- Neutrophil activating protein |  |  |
| Case +/- | 18/56 | 34/71 |
| Control +/- | 33/114 | 65/145 |
| *Unadjusted* | 1.09 (0.58, 2.06) | 1.08 (0.63, 1.84) |
| *Fully Adjusted* | 1.43 (0.70, 2.91) | 1.26 (0.65, 2.46) |
| HP0305- Hypothetical protein |  |  |
| Case +/- | 21/53 | 37/68 |
| Control +/- | 26/121 | 78/132 |
| *Unadjusted* | 1.83 (0.94, 3.56) | 0.91 (0.53, 1.54) |
| *Fully Adjusted* | **2.21 (1.04, 4.69)** | 0.93 (0.46, 1.88) |
| HpaA- Neuraminyllactose-binding hemagglutinin homolog |  |  |
| Case +/- | 20/54 | 36/69 |
| Control +/- | 34/113 | 70/140 |
| *Unadjusted* | 1.22 (0.64, 2.31) | 1.05 (0.63, 1.74) |
| *Fully Adjusted* | 1.31 (0.63, 2.74) | 1.40 (0.70, 2.82) |
| Cag delta- Cag island protein 3 |  |  |
| Case +/- | 25/49 | 37/68 |
| Control +/- | 34/113 | 63/147 |
| *Unadjusted* | 1.65 (0.91, 2.99) | 1.28 (0.77, 2.14) |
| *Fully Adjusted* | 1.85 (0.93, 3.67) | 1.33 (0.68, 2.57) |
| CagM- Cag island protein 16 |  |  |
| Case +/- | 24/50 | 49/56 |
| Control +/- | 38/109 | 96/114 |
| *Unadjusted* | 1.34 (0.74, 2.42) | 1.04 (0.64, 1.68) |
| *Fully Adjusted* | 1.22 (0.60, 2.46) | 0.81 (0.43, 1.55) |
| CagA |  |  |
| Case +/- | 25/49 | 46/59 |
| Control +/- | 32/115 | 62/148 |
| *Unadjusted* | 1.96 (1.02, 3.76) | 1.96 (1.17, 3.28) |
| *Fully Adjusted* | **2.16 (1.03, 4.50)** | 1.96 (0.98, 3.93) |
| HyuA- Hydantoin utilization protein A |  |  |
| Case +/- | 23/51 | 82/128 |
| Control +/- | 35/112 | 39/66 |
| *Unadjusted* | 1.43 (0.78, 2.61) | 0.92 (0.57, 1.49) |
| *Fully Adjusted* | 1.54 (0.79, 2.98) | 0.93 (0.50, 1.74) |
| Catalase- Detoxification |  |  |
| Case +/- | 22/52 | 43/62 |
| Control +/- | 34/113 | 82/128 |
| *Unadjusted* | 1.34 (0.74, 2.43) | 1.09 (0.66, 1.80) |
| *Fully Adjusted* | 1.49 (0.76, 2.90) | 0.92 (0.48, 1.77) |
| VacA- Vacuolating cytotoxin |  |  |
| Case +/- | 29/45 | 54/51 |
| Control +/- | 54/93 | 101/109 |
| *Unadjusted* | 1.11 (0.61, 2.01) | 1.16 (0.71, 1.88) |
| *Fully Adjusted* | 1.19 (0.61, 2.32) | 0.85 (0.45, 1.64) |
| HcpC- Conserved hypothetical secreted protein |  |  |
| Case +/- | 25/49 | 47/58 |
| Control +/- | 39/108 | 88/122 |
| *Unadjusted* | 1.48 (0.78, 2.81) | 1.14 (0.69, 1.88) |
| *Fully Adjusted* | 1.99 (0.94, 4.20) | 1.15 (0.60, 2.20) |
| Cad- Cinnamyl alcohol dehydrogenase ELI3-2 |  |  |
| Case +/- | 21/53 | 33/72 |
| Control +/- | 31/116 | 66/144 |
| *Unadjusted* | 1.47 (0.77, 2.79) | 1.00 (0.58, 1.73) |
| *Fully Adjusted* | 1.53 (0.71, 3.32) | 0.64 (0.30, 1.36) |
| Omp- Outer membrane protein |  |  |
| Case +/- | 26/48 | 51/54 |
| Control +/- | 52/92 | 98/112 |
| *Unadjusted* | 1.00 (0.55, 1.83) | 1.09 (0.66, 1.78) |
| *Fully Adjusted* | 0.99 (0.49, 2.00) | 0.95 (0.50, 1.79) |

^1^ *H. pylori* seropositivity is defined here as positivity to ≥ 4 *H. pylori* antigens
